# The impact of dietary inflammation index on gynecological and breast cancer risk in adult smoking women in the United States: A cross-sectional study based on NHANES data from 2007 to 2020

**DOI:** 10.1097/MD.0000000000049301

**Published:** 2026-06-26

**Authors:** Jifeng Li, Yifei Zeng, Dongxiao Zhang, Xiaohua Pei

**Affiliations:** a Beijing University of Chinese Medicine, Beijing, China; b The Third Affiliated Hospital, Beijing University of Chinese Medicine, Beijing, China; c Beijing Hospital of Traditional Chinese Medicine, Capital Medical University, Beijing, China.

**Keywords:** breast cancer, Dietary Inflammatory Index, gynecologic cancer, NHANES, smoking

## Abstract

Gynecologic and breast cancers (GBC) represent one of the most prevalent malignancies among women globally, and the Dietary Inflammation Index (DII) may influence its associated risk. This research aims to investigate the impact of DII on the risk of GBC in adult female smokers in the United States, utilizing National Health And Nutrition Examination Survey data from 2007 to 2020. A descriptive analysis was initially conducted to evaluate the dataset, followed by a binomial logistic regression model to assess the relationship between DII and GBC. Three models were developed: Model I (unadjusted), Model II (adjusted for age, race, education level, and income ratio), and Model III (further adjusted for confounding variables including body mass index, chronic diseases, alcohol consumption, and physical activity). A multifaceted sensitivity analysis was performed to validate the robustness of the findings. The analysis included 7501 participants, of whom 1291 were smokers. Results indicated that a higher DII was significantly associated with increased GBC risk. In Model I, each unit increase in DII corresponded to a 24% increase in risk (Odds ratios [OR]: 1.24, confidence intervals [CI]: 1.07–1.44, *P* = .005). Model II showed a 32% increase (OR: 1.32, CI: 1.13–1.55, *P* = .001), while Model III indicated a 27% increase (OR: 1.27, CI: 1.05–1.53, *P* = .017). Sensitivity analysis revealed no significant effects of DII on former smokers and nonsmokers (*P* > .05). Additionally, subgroup analysis based on race, education level, income, body mass index, and DII quartiles did not yield significant results (*P* > .05). Restricted cubic splines analysis did not identify a nonlinear relationship between DII and GBC (*P* = .307). The DII is significantly associated with the risk of GBC among female smokers, underscoring the necessity of incorporating dietary factors into cancer prevention and intervention strategies.

## 1. Introduction

Breast cancer (BC) and gynecologic cancers (which include uterine, cervical, and ovarian cancers) are among the most prevalent malignancies in women worldwide, profoundly impacting health and quality of life. The incidence of these malignancies, collectively referred to as gynecologic and BC (GBC), is rising globally, particularly in regions where lifestyles and diets are becoming increasingly Westernized.^[1]^ These cancers, especially breast and uterine cancers, often have multifactorial etiologies involving complex interactions between environmental and lifestyle factors, with diet and smoking identified as critical risk contributors.^[2–4]^

The Dietary Inflammatory Index (DII) has emerged as a crucial tool in cancer risk research in recent years, serving as a measure of the inflammatory potential of an individual’s diet by quantifying its pro-inflammatory and anti-inflammatory components. Given the established role of chronic inflammation in the pathogenesis and progression of various cancers,^[5]^ the DII has been increasingly utilized to evaluate dietary influences on cancer risk. High-DII scores have been linked to an elevated risk of several cancers, such as breast, colorectal, and lung cancers.^[6,7]^ However, there is a relative paucity of studies focusing on the risk of GBC among women who smoke. Smoking, a well-documented risk factor for multiple cancers, including lung, oral, and bladder cancers, is thought to exacerbate the risk of GBC by promoting immunosuppression, chronic inflammation, and hormonal imbalances.^[8,9]^

Given the heightened risk profile of female smokers, understanding the potential influence of the DII on GBC risk in this specific population remains a critical yet underexplored area. Therefore, using data from the United States (US) National Health and Nutrition Examination Survey (NHANES) spanning 2007 to 2020, this study seeks to explore the relationship between DII and the risk of GBC among female smokers. By elucidating this relationship, we hope to provide valuable insights for early cancer prevention strategies targeted at this high-risk group.

## 2. Methods

### 2.1. Data source

This cross-sectional study drew upon data from the NHANES database. Given that vitamin D data were unavailable prior to 2007, data from 7 consecutive cycles (2007–2020) were selected for inclusion. The study applied the following exclusion criteria: male participants; individuals under 20 years of age; current pregnancy; missing information on GBC diagnosis; incomplete data related to the DII; missing smoking-related information; and missing data on relevant covariates. After applying these criteria, eligible participants were included for subsequent analysis.

The NHANES data utilized in this study were publicly accessible via the NHANES website (https://www.cdc.gov/nchs/nhanes/). As this research involved a secondary analysis of publicly available data, further institutional review board approval was unnecessary.^[10]^

### 2.2. DII

In this study, we calculated the DII based on 28 dietary components, including cholesterol, various types of fatty acids (saturated, monounsaturated, polyunsaturated, n-3, and n-6), and essential nutrients such as niacin, retinol, thiamin, riboflavin, pyridoxine, cobalamin, ascorbic acid, vitamin D, tocopherol, iron, magnesium, zinc, selenium, folic acid, and β-carotene. Additionally, carbohydrates, protein, total fat, alcohol, fiber, caffeine, and energy were included. Nutrient intake was estimated using the average of 2 reliable 24-hour dietary recall interviews conducted by NHANES experts. To enhance the robustness of the data, we only included DII data from participants with 2 reliable 24-hour dietary recall records. The DII calculation followed the formula established in prior research.^[11]^

### 2.3. GBC

We defined GBC using the cancer section of the “Medical Conditions” questionnaire from the NHANES database. Participants were first asked, “Have you ever been told you have cancer or a malignancy?” Those who responded “yes” were then asked to specify the type of cancer. Respondents who reported only breast, uterine, cervical, or ovarian cancer as their primary and sole tumor were classified as GBC cases. Participants with a history of multiple cancers were excluded from this classification. In this study, BC was analyzed together with gynecologic cancers because these malignancies share common hormonal and reproductive risk factors and are often evaluated together in epidemiological studies focusing on women-specific cancers.^[12]^ Finally, individuals who answered “no” to having cancer were classified as non-GBC cases.

### 2.4. Smoking status

Participants were categorized into 3 groups based on their smoking status using smoking-related questions from the NHANES questionnaire: never smokers, former smokers, and current smokers. The definitions for each group are as follows: current smokers: participants who reported smoking “every day” or “some days” and had smoked at least 100 cigarettes in their lifetime; former smokers: participants who reported that they “no longer smoke” but had previously smoked at least 100 cigarettes in their lifetime; nonsmokers: participants who had smoked fewer than 100 cigarettes in their lifetime or had never smoked.

### 2.5. Covariates

Multiple potential covariates were included in the analysis, covering demographic characteristics, economic and educational status, alcohol consumption history, body mass index (BMI), female reproductive health status, and chronic disease status. Specifically, age was categorized into 2 groups: 20 to 50 years and over 50 years. Race was classified into non-Hispanic White, non-Hispanic Black, Mexican American, other Hispanic, and other ethnic groups (including Asian, other ethnicities, and multiracial individuals). Economic status was defined using the poverty-to-income ratio (PIR) and divided into 3 groups: low income (PIR < 1.0), middle income (PIR 1.0–4.0), and high income (PIR ≥ 4.0). Educational attainment was categorized into 3 levels: less than high school, high school graduate, and more than high school. Alcohol consumption was classified based on whether the individual consumed alcohol (yes/no). BMI was divided into 3 categories: < 25, 25–30, and > 30. Physical recreational activity levels were based on self-reports and categorized as no activity, moderate activity, or vigorous activity. Reproductive health-related covariates included age at menarche (< 12 years, ≥ 12 years), pregnancy history (yes/no), and female hormone use (yes/no). Chronic disease variables, including hypertension, diabetes, heart disease (coronary heart disease, heart failure, and angina), and stroke, were categorized into 2 groups based on the results of the NHANES questionnaire and laboratory examinations.

## 3. Statistical analysis

Descriptive statistical analyses were conducted following Center for Disease Control and Prevention guidelines. Continuous variables were presented as mean ± standard deviation (mean ± standard deviation), while categorical variables were expressed as counts and weighted percentages. The chi-square test was employed for comparisons of categorical variables. For continuous variables, the appropriate statistical test was chosen based on the results of the normality test. Normally distributed data were analyzed using Analysis of Variance, whereas the Kruskal–Wallis test was applied to data with skewed distributions. To assess the impact of DII on GBC risk among smoking women, a weighted generalized linear model was developed. Three regression models were constructed: Model I, an unadjusted model without covariates; Model II, adjusted for age, race, education level, and PIR; and Model III, which further adjusted Model II for additional confounders, including BMI, chronic diseases (e.g., heart disease, hypertension, diabetes, and stroke), alcohol consumption, recreational activities, age at menarche, pregnancy history, and history of estrogen use.

To verify the robustness of our findings, a thorough sensitivity analysis was performed. First, we assessed the association between DII and GBC incidence across various smoking statuses. Subsequently, a stratified analysis was performed among female smokers based on race, education level, PIR, and BMI to further investigate the impact of DII on GBC risk within various subgroups. Next, the continuous DII variable was converted into quartiles after excluding extreme DII values among female smokers, and the DII was reevaluated using the weighted generalized linear model. Finally, restricted cubic splines were then applied to explore nonlinear relationships.

All statistical analyses took into account the complex sampling design and were conducted using R software (version 4.3.2; R Foundation for Statistical Computing, Vienna, Austria). The R packages employed included “dietaryindex,” “survey,” and “rcssci.” odds ratios and their corresponding 95% confidence intervals were calculated through logistic regression, with statistical significance determined at a threshold of *P* < .05.

## 4. Results

### 4.1. Study population and baseline characteristics

**Figure 1.**
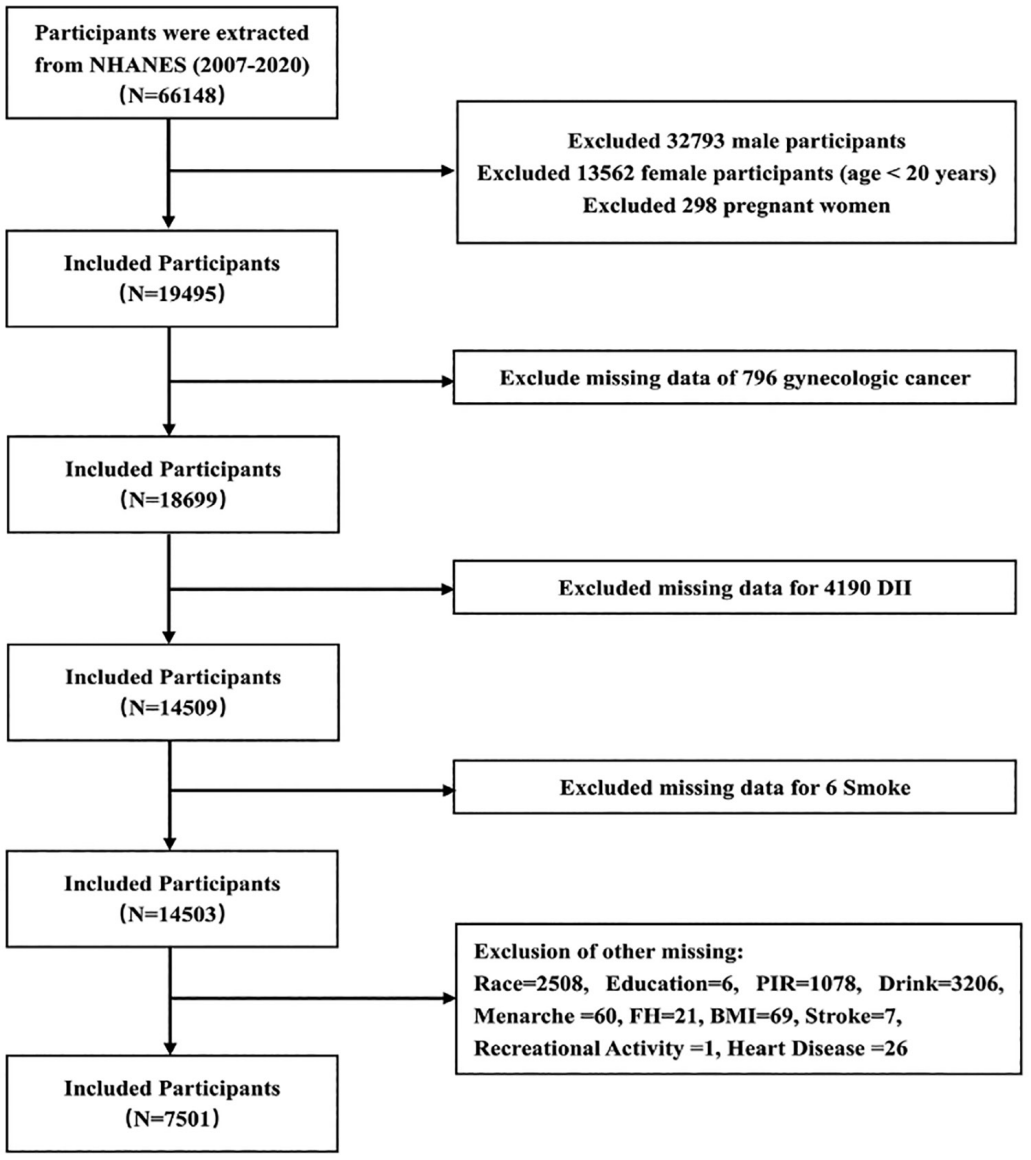
Study participant inclusion flowchart. A total of 66,148 participants were initially extracted from the NHANES dataset (2007–2020). The following exclusion criteria were applied: 32,793 male participants were excluded, along with 13,562 female participants under the age of 20 and 298 pregnant women. After these exclusions, 19,495 female participants remained. Additional exclusions were made for missing data related to gynecologic cancer (796 participants), followed by the exclusion of 4190 participants due to incomplete data on the DII. Subsequently, 14,503 participants were included, but further exclusions were made for missing smoking data (6 participants) and other missing variables (e.g., race, education level, physical activity, BMI, etc). The final cohort of 7501 participants was included in the study analysis, representing a total weighted population of 112,097,309. BMI = body mass index, DII = Dietary Inflammation Index, FH = female hormones, N = number of participants, NHANES = National Health and Nutrition Examination Survey.

Initially, 66,148 participants were assessed for inclusion in this study. Following the application of the exclusion criteria, a total of 7501 participants (reflecting a total weighted population of 112,097,309) were incorporated into the final analysis (Fig. 1).

The study population (Table 1) included smokers (n = 1291, 18.4%), former smokers (n = 1443, 19.8%), and nonsmokers (n = 4807, 61.8%). There were significant differences in DII scores across different smoking statuses (*P* < .001). Current smokers exhibited the highest mean DII score (2.03 ± 0.07), followed by former smokers (1.17 ± 0.08), while nonsmokers had the lowest mean DII score (1.24 ± 0.05). With respect to GBC, smoking status was significantly linked to the incidence of GBC (*P* < .001). Furthermore, variations in smoking status were significantly related to race, education level, income, alcohol consumption, BMI, recreational activities, pregnancy history, oral estrogen use, and history of hypertension, heart disease, and stroke (*P* < .05).

**Table 1:** Characteristics of survey participants included in the analysis, NHANES, 2007 to 2020.

Characteristics	Overall n = 7501	Smokers n = 1291 (18.4)	Former smokers n = 1403 (19.8)	nonsmokers n = 4807 (61.8)	*P* value
DII	1.37 ± 0.05	2.03 ± 0.07	1.17 ± 0.08	1.24 ± 0.05	< .001
GBC (n, %)					< .001
Yes	387 (4.9)	79 (7.1)	112 (7.6)	196 (3.4)	
No	7114 (95.1)	1212 (92.9)	1291 (92.4)	4611 (96.6)	
Age (n, %)					< .001
20–50	4095 (59.0)	807 (65.5)	518 (42.2)	2770 (62.4)	
> 50	3406 (41.0)	484 (34.5)	885 (57.8)	2037 (37.6)	
Race (n, %)					< .001
Hispanic American	1072 (8.1)	98 (4.6)	154 (5.4)	820 (10.1)	
Other Hispanic	842 (5.7)	104 (3.8)	145 (4.3)	593 (6.7)	
Non-Hispanic White	3074 (66.6)	676 (70.9)	750 (78.3)	1648 (61.6)	
Non-Hispanic Black	17,728(11.9)	327 (13.4)	268 (7.4)	1133 (13.0)	
Other race	785 (7.6)	86 (7.4)	86 (4.5)	613 (8.6)	
Education (n, %)					< .001
< High school	1598 (13.9)	357 (21.2)	280 (12.5)	961 (12.2)	
High school	1628 (21.5)	337 (33.4)	326 (21.4)	925 (18.0)	
> High school	4275 (64.6)	557 (45.4)	797 (66.1)	2921 (69.8)	
PIR (n, %)					< .001
< 1.0	1721 (16.1)	471 (28.7)	264 (11.6)	986 (13.9)	
1.0 ≤ PIR < 4.0	1835 (50.1)	671 (53.4)	736 (49.5)	2538 (49.3)	
≥ 4.0	3945 (33.8)	149 (17.9)	403 (38.9)	1283 (36.8)	
Drink (n, %)					< .001
Yes	4541 (67.1)	1021 (82.5)	1059 (81.0)	2461 (58.1)	
No	2960 (32.9)	270 (17.5)	344 (19.0)	2346 (41.9)	
BMI (kg/m^2^) (n, %)					.029
< 25	2171 (32.0)	416 (34.6)	328 (25.9)	1427 (33.1)	
25–30	2124 (28.5)	337 (27.4)	419 (30.8)	1368 (28.1)	
> 30	3206 (39.5)	538 (38.1)	656 (43.2)	2012 (38.8)	
RA (n, %)					< .001
Moderate	2158 (30.9)	323 (28.3)	451 (33.1)	1384 (31.0)	
Vigorous	1425 (24.4)	148 (13.7)	226 (23.7)	1051 (27.8)	
No	3918 (44.7)	820 (58.0)	726 (43.2)	2372 (41.1)	
Menarche (n, %)					.130
≤12	3592 (47.2)	640 (49.4)	659 (43.6)	2293 (47.7)	
>12	3609 (52.8)	651 (50.6)	744 (56.4)	2514 (52.3)	
Pregnant (n, %)					< .001
Yes	6327 (80.1)	1136 (86.3)	1255 (86.1)	3846 (76.4)	
No	1264 (19.9)	155 (13.7)	148 (13.9)	961 (23.6)	
FH (n, %)					< .001
Yes	1386 (19.8)	194 (16.2)	394 (29.6)	798 (17.8)	
No	6115 (80.2)	1091 (83.8)	1009 (70.4)	4009 (82.2)	
Diabetes (n, %)					< .001
Yes	981 (9.5)	136 (15.4)	256 (9.2)	211 (15.4)	
No	6520 (90.5)	1155 (84.6)	1147 (90.8)	58 (84.6)	
HD (n,%)					.001
Yes	395 (4.4)	82 (7.6)	113 (14.3)	598 (8.5)	
No	7106 (95.6)	1209 (92.4)	1290 (85.7)	4218 (91.5)	
Hypertension (n, %)					< .001
Yes	2730 (30.3)	460 (29.8)	648 (37.8)	1622 (28.1)	
No	4771 (69.7)	831 (70.2)	755 (62.2)	3185 (71.9)	
Stroke (n, %)					.004
Yes	252 (2.8)	68 (4.1)	63 (3.5)	123 (2.1)	
No	7249 (97.2)	1225 (95.9)	1340 (96.5)	4684 (97.9)	

Since the DII was a continuous variable (expressed as mean ± SD), and the data did not follow a normal distribution, ANOVA was applied. All other variables were categorical (expressed as n, weighted percentage), for which the chi-square test was used.

ANOVA = analysis of variance, BMI = body mass index, DII = Dietary Inflammatory Index, FH = female hormones, GBC = gynecologic or breast cancers, HD = heart disease, kg = kilogram, m = meter, n = number of participants, NHANES = National Health and Nutrition Examination Survey,.PIR = poverty-to-income ratio, RA = recreational activities, SD = standard deviation.

### 4.2. Relationship between DII and GBC in female smokers

Table 2 illustrates the relationship between DII and the incidence of GBC. In the unadjusted model (Model I), each 1-unit increase in DII corresponded to a significant 24% rise in the incidence of GBC. In Model II, after adjusting for variables such as age, race, education level, and PIR, the incidence of GBC increased by 32% for every 1-unit increase in DII score. In Model III, which further included covariates such as BMI and physical activity, each unit increase in DII was linked to a 27% increase in GBC risk. These findings indicate that higher DII scores are consistently and positively linked to an increased incidence of GBC among female smokers, even after considering potential confounding variables.

**Table 2:** WGLM analysis of DII (continuous variable) correlation between smoking women and GBC.

Model	OR	*P* value	95% CI
Model 1	1.24	.005	1.07–1.44
Model 2	1.32	.001	1.13–1.55
Model 3	1.27	.017	1.05–1.53

Model 1 AIC: 640.91; Model 2 AIC: 633.46; Model 3 AIC: 640.85.

The VIF of model 2 was < 10, indicating that there was no serious collinearity problem. However, there were 2 VIF >10 in model 3 (Recreational Activities [16.84] and HD [10.26]), so we recalculated Model 3 after excluding these 2 variables, and found *P* = .012(OR = 1.28; 95%CI: 1.04–1.57), so the results of model 3 above were still used.

AIC = Akaike information criterion, CI = confidence interval, DII = Dietary Inflammatory Index, GBC = gynecologic or breast cancers, HD = heart disease, OR = odds ratio, VIF = variance inflation factor, WGLM = weighted generalized linear model.

### 4.3. Sensitivity analysis

**Figure 2.**
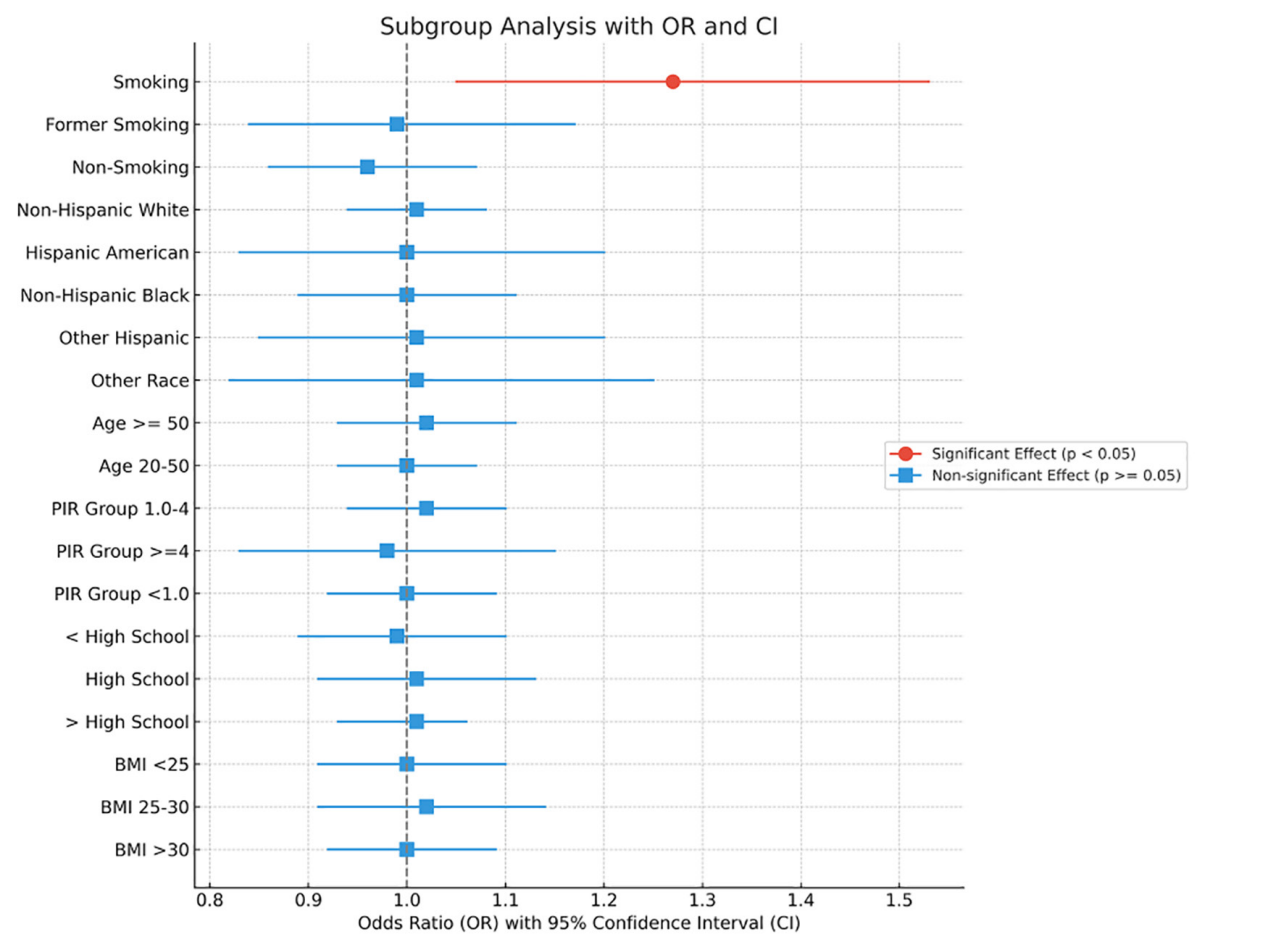
Subgroup analysis of the relationship between the DII and GBC incidence among female smokers. The plot shows the ORs with 95% CIs for various subgroups, including smoking status, race, age, socioeconomic status (PIR), education level, and BMI. Significant effects (*P* < .05) are indicated by red circles, while nonsignificant effects (*P* ≥ .05) are marked with blue squares. The analysis revealed that DII was not significantly correlated with GBC incidence across these subgroups. BMI = body mass index, CI = confidence interval, DII = Dietary Inflammatory Index, GBC = gynecologic and breast cancers, OR = odds ratio, PIR = poverty-to-income ratio.

**Figure 3.**
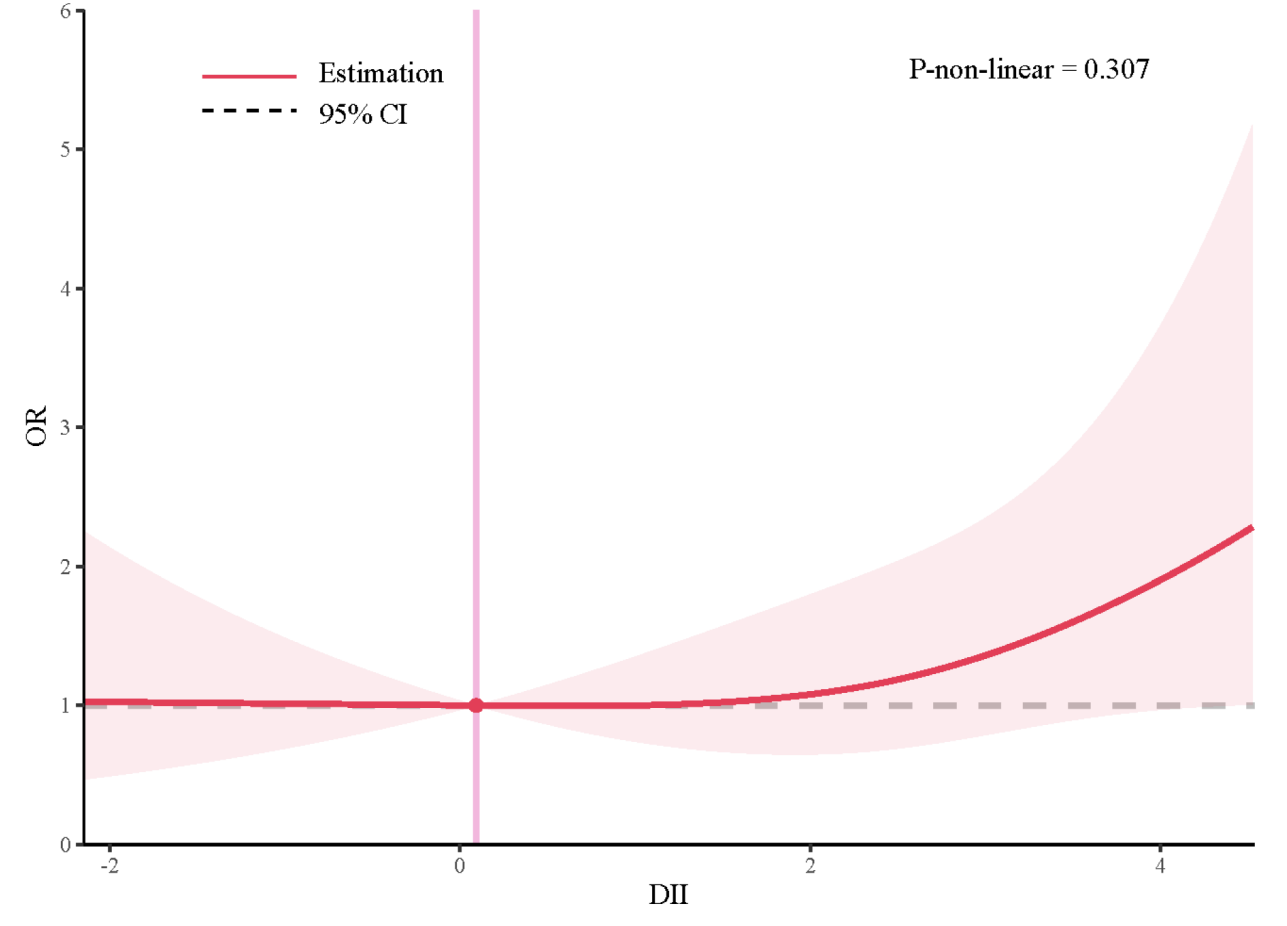
RCS analysis of the relationship between the DII and GBC in female smokers. The plot shows the OR with a 95% CI for DII, with the red line representing the estimated OR and the shaded region indicating the 95% CI. The analysis did not identify a significant nonlinear relationship between DII and GBC, as indicated by the *P* value for nonlinearity (*P* = .307). CI = confidence interval, DII = Dietary Inflammatory Index, GBC = gynecologic and breast cancers, OR = odds ratio, RCS = restricted cubic splines

The sensitivity analysis, which categorized participants according to their smoking status, showed that DII was strongly associated with an increased incidence of GBC among female smokers (*P* < .05). However, this association was not significant for former smokers and nonsmokers (*P* > .05). Additionally, further subgroup analysis among women who smoke revealed that DII was not significantly correlated with GBC incidence across various categories, including age, race, education level, income, or BMI (*P* > .05, Fig. 2). Although an increasing trend in odds ratios was observed across different DII quartiles, none of these associations reached statistical significance (*P* > .05), suggesting that the relationship between DII quartile variables and GBC risk in smoking women was not evident (Table S1, Supplemental Digital Content 1). Additionally, the restricted cubic splines analysis did not identify a significant nonlinear relationship between DII and GBC in smoking women (*P* = .307, Fig. 3).

## 5. Discussion

Utilizing NHANES data from 2007 to 2020, this study aimed to evaluate the association between DII and the risk of GBC in adult smoking women in the US. The findings demonstrate a strong positive correlation between DII and the likelihood of GBC, indicating that a higher dietary inflammatory potential is linked to an increased incidence of GBC among female smokers. This underscores the critical role of dietary factors in assessing GBC risk, particularly within the high-risk population of female smokers.

Further analysis indicated that when treated as a continuous variable, the DII was strongly correlated with the likelihood of GBC among female smokers (*P* < .05). However, when DII was categorized into quartiles or tertiles, the association lost statistical significance (*P* > .05). This discrepancy may be attributed to information loss and diminished statistical power inherent in quantile analysis, which fails to adequately capture the progressive impact of DII on cancer risk.^[13]^ In contrast, the continuous variable analysis more precisely revealed a linear association between DII and the likelihood of cancer development. Consequently, future investigations should prioritize the use of continuous variable analysis methodologies to evaluate the potential influence of DII more comprehensively on GBC.

GBC, which include breast, uterine, ovarian, and cervical cancers, are shaped by various elements such as genetic predisposition, hormonal fluctuations, and lifestyle habits. Smoking is recognized as a significant risk factor for these cancers and may further exacerbate inflammatory responses within the body, thereby increasing cancer risk.^[5]^ As a modifiable external factor, dietary properties (whether anti-inflammatory or pro-inflammatory) can influence the onset and progression of cancer through the regulation of chronic inflammation. The DII functions as a widely recognized instrument for evaluating the inflammatory potential of dietary patterns, assigning scores that range from pro-inflammatory to anti-inflammatory based on the intake of specific foods and nutrients.^[14]^ Generally, diets rich in antioxidants, fiber, vitamins, and minerals are associated with anti-inflammatory effects,^[15,16]^ while those high in sugars, fats, carbohydrates, and processed foods tend to provoke pro-inflammatory responses.^[17,18]^ In the NHANES data set, dietary data contained 28 key nutrients that met the above criteria, so we selected these data to calculate the DII.

Smoking is a well-established carcinogenic factor, primarily contributing to cancer development by inducing oxidative stress and chronic inflammation. The oxidative stress caused by smoking results in cellular damage through the release of reactive oxygen species and nitrogen free radicals. These reactive species not only damage intracellular proteins, lipids, and DNA but also activate pro-inflammatory pathways, promoting cancer development.^[19]^ Specifically in women, smoking-induced oxidative stress enhances the risk of estrogen-related cancers by altering cytochrome P450 enzyme activity in the liver, which in turn modifies estrogen metabolism, leading to the production of carcinogenic metabolites such as 16α-hydroxyestrone.^[20]^ Moreover, the oxidative stress and inflammatory response triggered by smoking interact with the DII in the diet, amplifying the DII’s pro-inflammatory effects. A high-DII diet further elevates the risk of GBC by increasing systemic inflammation and exacerbating oxidative stress induced by smoking.

The results of this study align with previous research,^[21–23]^ while also offering unique contributions to the existing literature. While earlier studies have primarily concentrated on the connection between the DII and cancer occurrence in the general population, this research is the first to specifically examine how the DII influences GBC development among adult women who smoke. Smoking not only exacerbates the burden of inflammation within the body but also interacts synergistically with unhealthy dietary patterns, further elevating the risk of GBC. This finding underscores the importance of public health interventions targeting smoking women, emphasizing that efforts should not only focus on smoking cessation but also prioritize improving dietary habits to reduce the inflammatory potential of their diets, thereby mitigating cancer risk.

While this research offers new insights into the relationship between the DII and GBC, it is imperative to consider its limitations. Firstly, the cross-sectional design precludes causal inference and cannot rule out the possibility of reverse causation, whereby a cancer diagnosis could alter dietary behaviors. Secondly, key confounding variables, such as menopausal status, were not available in the dataset and could not be adjusted for, potentially introducing residual confounding. Thirdly, the study relies on self-reported data for both the outcome (GBC) and exposure (smoking status), which are subject to recall and reporting biases and lack validation against medical records.

Fourthly, this study utilizes the NHANES dataset, which primarily reflects the health and nutrition status of US adult women. Therefore, the generalizability of the findings to other populations should be interpreted with caution, as dietary patterns and smoking behaviors may differ substantially across regions such as Asia and Europe. Finally, while an association was identified, the underlying biological mechanisms remain unexplored in this analysis. Therefore, future longitudinal studies are necessary to establish temporal relationships and causality. These studies should incorporate objective biomarker validation, including C-reactive protein and leukocytes, as potential mediators, and investigate specific biological pathways, such as inflammatory response and oxidative stress, to provide deeper insights into the mechanisms driving the observed associations.

Public health interventions should be tailored in response to these findings. For smoking women, it is crucial not only to encourage smoking cessation but also to provide individualized dietary recommendations that promote the intake of antioxidant-rich foods (such as fruits, vegetables, and nuts) while reducing the consumption of foods high in sugar, fat, and processed ingredients. An integrated strategy that combines smoking cessation with dietary changes may significantly reduce the likelihood of GBC in this high-risk group.

In summary, this study offers new insights into the relationship between DII and GBC risk among women who smoke, highlighting the potential for dietary modifications as a strategy for cancer risk reduction. However, the cross-sectional nature of the study limits the ability to infer causality. Future research, particularly randomized controlled trials, is essential to establish the causal relationship between DII and GBC risk. Such studies would provide more robust evidence to inform the development of more effective public health intervention strategies targeting both smoking cessation and dietary improvements.

## 6. Conclusion

This study ultimately sheds light on how dietary inflammation affects the risk of GBC in women who smoke, potentially influencing the creation of public health policies and tailored nutritional interventions.

## Acknowledgments

The author would like to extend heartfelt appreciation to the staff and participants of the NHANES study for their significant contributions.

## Author contributions

**Conceptualization:** Jifeng Li, Dongxiao Zhang, Xiaohua Pei.

**Data curation:** Jifeng Li, Dongxiao Zhang, Xiaohua Pei.

**Formal analysis:** Jifeng Li.

**Funding acquisition:** Dongxiao Zhang, Xiaohua Pei.

**Investigation:** Jifeng Li, Yifei Zeng, Dongxiao Zhang, Xiaohua Pei.

**Methodology:** Jifeng Li, Yifei Zeng, Dongxiao Zhang, Xiaohua Pei.

**Project administration:** Dongxiao Zhang, Xiaohua Pei.

**Resources:** Dongxiao Zhang, Xiaohua Pei.

**Supervision:** Dongxiao Zhang, Xiaohua Pei.

**Validation:** Jifeng Li, Yifei Zeng, Xiaohua Pei.

**Visualization:** Xiaohua Pei.

**Writing – original draft:** Jifeng Li, Dongxiao Zhang, Xiaohua Pei.

**Writing – review & editing:** Jifeng Li, Yifei Zeng, Dongxiao Zhang, Xiaohua Pei.


