# Supplementary material for: The impact of dietary inflammation index on gynecological and breast cancer risk in adult smoking women in the United States: A cross-sectional study based on NHANES data from 2007 to 2020
Source: Medicine (Baltimore). 2026 Jun 26;105(26):e49301. doi: 10.1097/MD.0000000000049301 (PMC13313734; doi:10.1097/MD.0000000000049301)
Supplement: Supplementary file 1 [file medi-105-e49301-s001.docx]

Supplementary Table 1 WGLM analysis of DII (categorica variable) correlation between smoking women and gynecological and breast cancers

| Quartile | OR | P-value | 95% CI |
| --- | --- | --- | --- |
| Q1 | 1.00 |  |  |
| Q2 | 1.40 | 0.58 | 0.44-4.30 |
| Q3 | 2.32 | 0.13 | 0.85-6.42 |
| Q4 | 1.95 | 0.25 | 0.60-6.38 |
